# Reoccurrence of botulinum neurotoxin subtype A3 inducing food-borne botulism, Slovakia, 2015

**DOI:** 10.2807/1560-7917.ES.2017.22.32.30591

**Published:** 2017-08-10

**Authors:** Lucia Mad’arová, Brigitte G Dorner, Lars Schaade, Vladimír Donáth, Mária Avdičová, Milota Fatkulinová, Jozef Strhársky, Ivana Sedliačiková, Cyril Klement, Martin B Dorner

**Affiliations:** 1 Regional Authority of Public Health Banská Bystrica, Banská Bystrica, Slovakia; 2 Robert Koch Institute, Consultant laboratory for neurotoxin-producing clostridia (botulism, tetanus), Berlin, Germany; 3 Robert Koch Institute, Centre for Biological Threats and Special Pathogens, Berlin, Germany; 4 F. D. Roosevelt Teaching Hospital, Department of Neurology, Slovak Medical University, Banská Bystrica, Slovakia; 5 Slovak Medical University, Faculty of Public Health, Bratislava, Slovakia

**Keywords:** Botulism, food-borne, *Clostridium botulinum*, subtype A3, hummus, commercial

## Abstract

A case of food-borne botulism occurred in Slovakia in 2015. *Clostridium botulinum* type A was isolated from three nearly empty commercial hummus tubes. The product, which was sold in Slovakia and the Czech Republic, was withdrawn from the market and a warning was issued immediately through the European Commission’s Rapid Alert System for Food and Feed (RASFF). Further investigation revealed the presence of botulinum neurotoxin (BoNT) subtype BoNT/A3, a very rare subtype implicated in only one previous outbreak (Loch Maree in Scotland, 1922). It is the most divergent subtype of BoNT/A with 15.4% difference at the amino acid level compared with the prototype BoNT/A1. This makes it more prone to evading immunological and PCR-based detection. It is recommended that testing laboratories are advised that this subtype has been associated with food-borne botulism for the second time since the first outbreak almost 100 years ago, and to validate their immunological or PCR-based methods against this divergent subtype.

## Introduction

The life-threatening illness botulism is caused by seven serotypes (A-G) of botulinum neurotoxins (BoNT), however only serotypes A, B, E and F cause disease in humans. BoNTs prevent neurotransmitter release and lead to a flaccid paralysis. There are three main forms of human botulism: food-borne, wound and infant botulism [1,2]. The BoNTs are produced by six phylogenetically and physiologically distinct bacteria (*Clostridium botulinum* Groups I–IV, and strains of *C. baratii* and *C. butyricum*) which are so diverse as to merit classification as different species. All of the BoNT-producing *Clostridium* species have non BoNT-producing counterparts (not harbouring the BoNT gene cluster) which are often assigned to different species names, such as *C. sporogenes* for *C. botulinum* Group I strains [2]. This polyphyly of *C. botulinum* together with its close relationship to non-toxigenic species can result either in the erroneous detection of toxigenic *Clostridium* species or failure thereof.

The BoNT gene cluster is often associated with mobile genetic elements, allowing for interconversion between toxigenic and nontoxigenic *Clostridium* species, and leading to substantial variation in BoNT sequences and their genomic backgrounds [3]. Today, the BoNT serotypes are divided into more than 40 subtypes, including BoNT/A1 to BoNT/A8 [4], and mosaic forms [5]. The BoNT diversity at the amino acid level can reach up to 36% within a serotype and is reflected by differences in their catalytic activity, receptor binding or pharmacokinetics [6]. In addition to containing the gene for BoNT, the BoNT gene cluster contains the gene for the non-toxic non-haemagglutinin (NTNH) protein. The BoNT gene cluster also harbours either three haemagglutinin (HA) genes (*ha1*, *ha2* and *ha3*) in the HA cluster or three *orfX* genes (*orfX1, orfX2* and *orfX3*) in the orfX cluster. Together with BoNT, these accessory proteins can form complexes of varying size and composition [7]. The variability observed in BoNTs due to the existence of seven serotypes, mosaic forms, several different subtypes and the occurrence of BoNT in different complexes, as well as their heterogeneous genetic background are probably the main reasons for the spectrum of clinical presentations and disease duration associated with botulism, and pose a tremendous challenge to diagnostic laboratories [8].

Botulism is associated with a wide variety of foods that are rich in protein and kept under the partial exclusion of oxygen, or in an environment where oxygen has been depleted by other bacteria. Marinated meat, fish, fruits, vegetables or mushrooms as well as home-canned products and home-cured or smoked meat or fish products, which are consumed unheated, are particularly known sources. BoNTs are heat-labile and can be destroyed by temperatures above 70 °C in minutes [9,10]. Nowadays, food-borne botulism is almost exclusively associated with home-produced foods, but commercial food products are occasionally involved in single cases or small outbreaks [11-13]. Between 2010–2014, ECDC reported 492 cases in Europe [14], with the last case occurring in Slovakia in 2012 [15].

### The event

In mid-August 2015 (day 0), an otherwise healthy adult in their 40s consumed, for dinner, three packs (100 g each) of a commercial hummus spread. The hummus, which was sold in Slovakia and the Czech Republic, had a best-before date of 9 days after consumption. The following morning (day 1), 9–11 hours after consumption of the hummus, the patient experienced nausea, vomiting, double vision, unsteady gait, dizziness, generalised weakness and difficulties swallowing, and was admitted to hospital. Despite receiving three doses of equine trivalent anti-A, B and E antitoxin, the patient felt into a coma on day 2 and was transferred to the intensive care unit (ICU). As botulism is a mandatory notifiable disease in Slovakia, the clinic reported the suspected botulism case to local health authorities. A gastric lavage, but neither serum nor stool, was taken on day 2 and stored at 4 °C for further analysis. After one week of storage, the gastric lavage was sent to the Public Health Institute in Ostrava, Czech Republic where it tested negative by mouse bioassay. Treatment consisted mainly of intensive symptomatic care and respiratory support for almost 50 days. The patient was discharged from hospital after 78 days, but still complained about breathing problems as of their last follow-up in May 2017.

## Methods

### Epidemiological investigation

On day 1, after being notified by the clinic, the regional health authorities started an epidemiological investigation by interviewing the hospitalised patient. The next day, day 2, the patient’s household was inspected to check for additional cases and to collect food samples. There, a close relative who shares the same household was interviewed, a list of food items consumed by the patient over the last days was recorded and suspicious food items were collected.

In cooperation with the Slovak State Veterinary and Food Administration (ŠVPS), an investigation on the composition and the origin of the ingredients used for production of the hummus was conducted at the food processing facility.

### Laboratory investigation

#### Isolation of *Clostridium botulinum* from hummus and molecular analysis

Small particles of hummus left on the packaging of the three nearly empty hummus tubes were streaked on tryptic soy agar and Schaedler agar plates, and subjected to anaerobic culture at 37 °C in anaerobic jars following EN ISO 7937:2005. Almost all colonies showed typical clostridia-like morphology and were probed for the presence of *bont* genes with a multiplex PCR for *bont/A*, *bont/B*, *bont/E* and *bont/F* [16] according to CEN ISO/TS 17919:2013.

#### Genetic analysis

Isolates were transferred to the Consultant laboratory for neurotoxin-producing clostridia (botulism, tetanus), formerly called the Consultant Laboratory for *Clostridium botulinum*, at the Robert Koch Institute (RKI) in Berlin, Germany for further molecular biological investigation. Isolates were grown in tryptone-peptone-glucose-yeast extract (TPGY) medium at 32 °C in an anaerobic workstation (MG500, Don Whitley, Shipley, United Kingdom), and tested for the presence of *bont/A–G* by single and multiplex quantitative PCR [17]. In addition, a quantitative PCR was used to test for the *ntnh* gene as a surrogate marker for BoNT-producing *Clostridium* spp. [18]. To further investigate the composition of the BoNT gene cluster, a set of six conventional PCRs targeting the three *ha* genes and the three *orfX* genes was applied [19]. Toxin production in supernatants was confirmed by in-house ELISA tests as described [20,21].

The 16S rRNA and *bont/A* gene sequences were obtained by Sanger sequencing [22]. Sequences were assembled and compared with the GenBank database at the National Center for Biotechnology Information in the United States via Geneious software packages (Biomatters Limited, Auckland, New Zealand) using the BLAST algorithm [23]. For subtyping, the amino acid sequence of the BoNT was compared with the eight known BoNT/A subtypes [4]. Sequences of the other BoNT/A subtypes were uploaded into Geneious and pairwise identities of the aligned sequences were calculated using the MAFFT algorithm [24]. For comparison, the BoNT subtypes of the following BoNT strains causing food-borne botulism in the past (except for BoNT/A4 which originated from an infant botulism case) were selected (GenBank accession numbers in parenthesis): BoNT/A1 (HA cluster) strain ATCC3502 (AM412317); BoNT/A1 (orfX cluster) strain NCTC2916 (X52066); BoNT/A2 (orfX cluster) strain Mascarpone (DQ310546); BoNT/A3 (orfX cluster) strain Loch Maree (CP000963); BoNT/A4 (orfX cluster) strain 657 (EU341307); BoNT/A5 (HA cluster) strain 1430.11 (KC683799); BoNT/A6 (orfX cluster) strain CDC41370 (FJ981696); BoNT/A7 (unknown cluster) strain 2008–148 (JQ954969); BoNT/A8 (orfX cluster) strain Chemnitz (KM233166).

#### Further microbiological investigation

In order to identify additional possibly contaminated products of the same production date, the Regional Authority of Public Health (RAPH) in Banská Bystrica investigated the products’ availability at several local supermarkets and the food bank. While no additional hummus was available from the food bank, three tubes of hummus with the same production date as the ones consumed were obtained from a supermarket. Unopened products were stored at 37 °C for several days to reach suitable conditions for *C. botulinum*/*C. sporogenes* germination and growth.

## Results

### Epidemiological investigation

Results from the interview revealed that on day 0, the patient alone ate scrambled eggs for breakfast; the patient, a close relative and a friend shared the same meal of mushrooms in cream sauce with pasta for lunch; and the patient alone ate the hummus at dinner after obtaining it from a food bank. The interview of the patient and a close relative revealed that one tube of the hummus product showed gas production prior to being opened, but it tasted normal according to the patient. The epidemiologists notified the Regional Veterinary and Food Administration of the Slovak Republic and the three nearly empty tubes of hummus and two unopened packs of a commercial ready-to-eat sauerkraut and vegetarian sausage meal, from the same company as the hummus, were sent for laboratory investigation. Neither the scrambled eggs nor the pasta dish were available for sampling.

An investigation at the producing company revealed that the hummus product consisted of the following ingredients: chick peas, tofu, rapeseed oil, onion, salt, maize starch and spices. It also revealed that the chick peas originated from Brazil or Argentina.

### Laboratory investigation

RAPH received the collected food items, including the three opened and nearly empty 100 g hummus tubes. Three strains with typical *C. botulinum* morphology (designated Banska Bystrica, RAPH1223j and RAPH1224) were isolated, one from each tube. Two of them, Banska Bystrica and RAPH1223j, were PCR-positive for the *bont/A* gene. The vegetarian sausage and sauerkraut meal tested negative for the presence of *C. botulinum* and the *bont/A* gene. On day 7, as soon as the first results were available, the hummus was withdrawn from the market and a Food and Feed Safety Alert was issued through the European Commission’s Rapid Alert System for Food and Feed (RASFF).

The three unopened hummus tubes with the same production date that were obtained from a supermarket were subjected to 37 °C storage. Microbiological analysis and PCR testing did not reveal the presence of *C. botulinum* or *C. sporogenes* despite some signs of gas formation during storage.

#### Molecular biological characterisation of isolated strains

The sequences of the 16S rRNA gene analysed at RKI revealed that all three isolates (strains Banska Bystrica, RAPH1223j and RAPH1224) belonged to *C. botulinum* Group I/*C. sporogenes* (> 99% identity), which are almost indistinguishable at the 16S rRNA sequence level [2,25]. The presence of *bont/A* and *ntnh* genes was confirmed by quantitative PCR, and expression of the toxin was shown by an in-house ELISA for isolates Banska Bystrica and RAPH1223j. From the two BoNT/A-positive isolates, the BoNT/A sequences were obtained and compared with GenBank entries at the nucleotide (nt) and the amino acid level. Both toxigenic isolates (Banska Bystrica and RAPH1223j) were found to harbour a *bont/A* gene of identical sequence; the *bont/A* sequence of one strain, Banska Bystrica, was submitted to GenBank under accession number KU376389. It showed the highest identity (≥ 99.6%) to *bont/A* sequences of the unusual subtype BoNT/A3. Table 1 shows the distances computed against all BoNT/A subtypes at the nt and the amino acid levels.

**Table 1:** Percent identities at the nucleotide and amino acid levels of *Clostridium botulinum* Group I strain Banska Bystrica^a^ harbouring subtype BoNT/A3 compared with *C. botulinum* strains harbouring other BoNT/A subtypes^b^

Feature	Botulinum neurotoxin subtype								
	A1	A1(B)	A2	A3	A4	A5	A6	A7	A8
**Cluster type**	HA	orfX	orfX	orfX	orfX	HA	orfX	unknown	orfX
**Strain**	ATCC3502	NCTC2916	Mascarpone	Loch Maree	657	1430.11	CDC41370	2008–148	Chemnitz
**Nucleotide level**	92.0%	91.9%	96.3%	99.9%	91.5%	92.2%	92.8%	91.9%	93.1%
**Amino acid level**	84.6%	84.5%	92.9%	99.8%	84.3%	85.2%	86.3%	84.9%	87.7%

BoNT: botulinum neurotoxin.

^a^ As both of the BoNT/A-positive isolates had the identical BoNT/A sequence, only that of strain Banska Bystrica was submitted to GenBank under accession number KU376389.

^b^ Only one example for each subtype (from food-borne botulism, except for subtype A4, which originated from an infant botulism case) is selected and compared.

Compared with the prototype BoNT/A1, the BoNT of the new BoNT/A-positive isolates, Banska Bystrica and RAPH1223j, had an identity at the amino acid level of only 84.6% (compared with BoNT/A1 strain ATCC3502; HA cluster) or 84.5% (compared with BoNT/A1 strain NCTC2916; orfX cluster). Similar low amino acid level identities (between 84.3% and 87.7%) were found when compared with BoNT subtypes A4–A8, whereas amino acid level identity with subtype A2 (strain Mascarpone) was 92.9%. Sequence identities at the nt level were generally higher, but still diverged by up to 8.5%. Both isolates (Banska Bystrica and RAPH1223j) were negative for the *ha1, ha2* and *ha3* genes and positive for the *orfX1, orfX2* and *orfX3* genes by PCR; this is in agreement with the BoNT/A3 strain Loch Maree in which the *bont/A* gene is located in the orfX cluster.

## Discussion

Our laboratory investigations support the diagnosis of a case of food-borne botulism caused by the unusual BoNT subtype A3 in food consumed by the patient. To our knowledge, this is only the second time that this subtype was involved in food-borne botulism since eight people died from contaminated wild duck paste in Loch Maree, Scotland in 1922 [26]. No other strain belonging to this specific A3 subtype was isolated or subtyped for nearly a century until Lúquez and colleagues identified three strains from Argentina (in soil and salad) in 2012 [27]. Interestingly, the two described BoNT/A-positive isolates have an identical BoNT/A sequence at the amino acid level as one of the strains (CDC54054) isolated from Argentinian soil [27], and the chick peas used in the hummus consumed by the patient originated from South America, either from Brazil or Argentina.

Initially, suspected botulism could not be confirmed by testing the gastric lavage taken on day 2. Considering the acidity and proteolytic activity, as well as the length of storage, the gastric lavage taken after emesis on day 1 was not an ideal sample. Serum and stool samples, which have been proposed to be the most reliable samples for diagnostic testing [28], might not have been collected, because of a lack of testing facilities and inexperienced clinical personnel, particularly since botulism is a rare disease and no cases have been notified in Slovakia since 2012 [15]. For the clinician, it is important to consider the optimal timing when choosing samples for botulism diagnosis. Despite demonstration of BoNT in serum as one of the key tests to confirm botulism, the time period in which BoNT can be detected from serum is usually limited to the first few days after oral ingestion. However, confirmation of BoNT-producing *Clostridium* species (spores) from faeces can often be achieved over a more prolonged period (up to two weeks) [28,29]. Therefore, co-examination of serum and stool is recommended to confirm clinical cases of food-borne botulism [28-31]. The mere detection of *C. botulinum* from faeces without clinical symptoms does not imply the presence of botulism given that *C. botulinum* spores are not usually detected in the faeces of healthy humans. However, on rare occasions, spores that have been ingested with naturally contaminated food (e.g. honey, fresh vegetables) might be detected in the faeces of otherwise healthy people [28].

In our case, food-borne botulism could only be confirmed by the isolation of *C. botulinum* from two of the three consumed tubes of hummus. It is always hard to exclude that the analysed food become cross-contaminated in the household with *C. botulinum* by handling with kitchen utensils contaminated via another food. However, there was no indication that the hummus became cross-contaminated within the patient’s household. In the aftermath of the current botulism case, three unopened hummus tubes with the same production date were subjected to 37 °C storage and analysed for the presence of *C. botulinum* without any positive result. It is not unusual that *C. botulinum* cannot be detected from other items of the same lot. In three botulism outbreaks in Europe in 2011, which involved commercial olive tapenade, almond-stuffed olives and korma sauce, *C. botulinum* and BoNT could only be identified from one or two opened jars/pots obtained from the households, but not from any unopened jars/pots from the same lot (consisting of 60, 900, and 1,836 items, respectively) that were tested [11,32-34]. These cases indicate that even in modern industrialised food production, contamination of a single or a very few items within a lot cannot be completely excluded. In all those cases, consumer protection had utmost priority and food recalls were issued solely on the basis of positive results from the remaining product in the open containers from the affected households to prevent a potential spread of the disease [32-34].

Hummus has been involved in a food-borne botulism outbreak in the past, possibly due to incorrect storage temperature [35]. Whether this was the case in the event presented here could not be determined, but the patient did notice gas formation in the products. This might be indicative of spoilage as growth of *C. botulinum* in spoiled products is often associated with gas production, e.g. foaming, bulging of cans [36]. However, unlike Group II strains of *C. botulinum* which can grow at refrigeration temperatures, Group I strains (to which this study’s isolated strains belong) require growth temperatures above 10 °C [2]. This suggests that the hummus product was at least partially stored above refrigeration temperatures.

Compared with the known eight BoNT/A subtypes (BoNT/A1 to BoNT/A8 [4]), the subtype A3 is both very rare and the most distant from the prototype BoNT/A1 (Table 1), being up to 15.4% and 8% different at the amino acid and nt level, respectively. Notably, because of its amino acid sequence divergence, BoNT/A3 is known to be neutralised less efficiently by polyclonal antibodies generated against BoNT/A1 [37]. This has led us to speculate that the long lasting neurological effects observed in the patient despite the administration of three doses of trivalent antitoxin might at least be partially due to suboptimal neutralisation by the antitoxin, which is directed against BoNT/A1.

The rareness and divergence of the BoNT/A3 subtype poses the risk that it might not be detected by immunological or sequence-based diagnostic assays that have not been validated against it. In fact, one ELISA has already been shown to be unable to detect it [38]. Furthermore, in one [39] of the four mentioned PCR methods in ISO ISO/TS 17919:2013, the sequence of one primer and the probe each contain one mismatch towards all BoNT/A3 sequences. These mismatches might therefore hamper identification of strains belonging to the A3 subtype by the corresponding PCR [40,41]. Testing laboratories should be aware that strains harbouring this rare subtype can occur in European countries and are advised to validate their methods accordingly. Unfortunately, the exchange of BoNT-producing strains and in some instances, even the sharing of their sequence information are restricted because of their nature as possible biowarfare agents and other dual-use concerns [42,43].

## Conclusion

Rare and novel BoNT sequence variation can especially easily escape detection by sequence-based or immunological methods. As more sequences become available despite some restrictions, diagnostic facilities should check their PCRs frequently to ensure inclusion of all known sequence variations.

As a result of the rareness of botulism, many laboratories have no or only individual assays for detection at hand. Specialised laboratories (e.g. national reference laboratories or consultant laboratories) able to apply a broader range of methods could aid those laboratories, but are often not easily identifiable by the laboratories responsible for testing the samples.
